# Silicon Passivation by Ultrathin Hafnium Oxide Layer for Photoelectrochemical Applications

**DOI:** 10.3389/fchem.2022.859023

**Published:** 2022-03-25

**Authors:** Laurynas Staišiūnas, Putinas Kalinauskas, Eimutis Juzeliūnas, Asta Grigucevičienė, Konstantinas Leinartas, Gediminas Niaura, Sandra Stanionytė, Algirdas Selskis

**Affiliations:** Centre for Physical Sciences and Technology, Vilnius, Lithuania

**Keywords:** silicon, passivation, photoelectrochemistry, hafnium oxide, nanogravimetry, hydrogen photogeneration

## Abstract

Hafnium oxide (HfO_2_) films on silicon have the potential for application in photovoltaic devices. However, very little is known about the photoelectrochemical and protective properties of HfO_2_ films on Si. In this study, ultrathin films of HfO_2_ in the range of 15–70 nm were deposited on p-Si and Au substrates by atomic layer deposition (ALD). Grazing incidence X-ray diffraction (GI-XRD) identified the amorphous structure of the layers. Quartz crystal nanogravimetry (QCN) with Si and Au substrates indicated dynamics of electrolyte intake into the oxide film. No indications of oxide dissolution have been observed in acid (pH 3) and alkaline (pH 12) electrolytes. Mott–Schottky plots showed that the dark Si surface adjacent to the SiHfO_2_ interface is positively charged in an acid electrolyte and negatively charged in an alkaline electrolyte. The number of photoelectrons was determined to be much greater than the doping level of silicon. The cathodic photoactivity of the p-Si electrode protected by HfO_2_ films was studied with respect to the reaction of hydrogen reduction in acid and alkaline solutions. In acid solution, the film enhanced the reduction process when compared to that on the coating free electrode. The acceleration effect was explained in terms of prevention of silicon oxide formation, whose passivating capability is higher than that of hafnia films. In an alkaline electrolyte, an inhibition effect of the film was determined. Hafnia films protected Si from corrosion in this medium; however, at the same time, the film reduced electrode activity.

## Introduction

Thin films of metal oxides are promising materials for the protection of semiconductors from aggressive environments and improving of their stability. Electrochemical instability of silicon (Si) in electrolytes is an issue, which has impeded the development of sunlight-driven photovoltaic (PV) systems for water splitting (Hu et al., 2015). Several oxides have been studied for protection of p-Si photocathodes, for example, TiO_2_ (Seger et al., 2013), ZnO (Sun et al., 2012), SiO_x_ (Esposito et al., 2013; Choi et al., 2014), Al_2_O_3_ (Choi et al., 2014), and SrTiO_3_ (Ji et al., 2015). Hafnium oxide (hafnia, HfO_2_) is a potential candidate for Si protection due to its high chemical inertness, optical transparency, wide band gap, and high dielectric constant.

Passivation effects by hafnium oxide for efficiency enhancement in organic and inorganic solar cells have recently been reported (Polydorou et al., 2018; Cui et al., 2017b; Singh et al., 2016; Oudot et al., 2018). A *p*-type Si with a 15-nm hafnia film showed an effective surface recombination velocity of 9.9 cm s^−1^ and an interface state density of 3.6 × 10^10^ cm^−2^ eV^−1^ (Cui et al., 2017b). Efficiency of Si photocathodes with a HfO_2_ interlayer between a p^+^/n/n^+^ structure and a Ni_3_N/Ni cocatalyst was studied (Zhanget al. 2021). It was shown that a 1-nm HfO_2_ interlayer enhanced the electrode efficiency by 9%. A p-Si substrate was protected by a HfO_x_/SiO_x_ bilayer used in the hybridization of the photoelectrochemical (PEC) device with a thermoelectric device (Jung et al., 2019). Protection of Si surface nanostructures by HfO_2_ was also studied (Xing et al., 2017). The authors concluded that the HfO_2_ layer on nanoporous Si was less stable than the TiO_2_ layer.

Passivation in photovoltaics (PV) and electrochemistry refers to different, albeit related, phenomena. Electronic passivation in PV addresses reduction of interface state density (*D*_if_) (unsaturated bonds) on the semiconductor surface, for example, by chemical reduction applying passivating dielectrics (Cui et al., 2017a; Singh et al., 2016; Oudot et al., 2018). Another important type is the field effect passivation, which relates to electrostatic shielding of the charge carriers from the interface by an internal electric field, for example, films having a negative (Al_2_O_3_, SiO_2_+HfO_2_) or positive (SiN_x_) charge (Singh et al., 2016; Oudot et al., 2018).

Electrochemical passivation refers primarily to the inhibition of the charge transfer from semiconductor to electrolyte, or *vice versa*. To this end, the semiconductor is coated with a layer that protects the substrate from aggressive environments and prevents electron transfer to the electrolyte (Hu et al., 2015; Seger et al., 2013; Sun et al., 2012; Esposito et al., 2013; Choi et al., 2014; Ji et al., 2015). Another kind of passivation is the spontaneous formation of protective films from corrosion products (Hu et al., 2015). It is desirable for a photo-electrode that the passive layer can reduce recombination velocity, protect the Si–electrolyte junction from corrosion, and not inhibit substantially the electron transfer to electrolyte, that is, the layer does not reduce efficiency of (photo) electrochemical process. However, in most cases, oxide films mitigate Si corrosion at the expense of surface activity: the electrode becomes electrochemically inactive in certain potential regions. The dilemma is typically solved in favor of passivity because dissolution leads to continual destruction of the semiconductor and failure propagation throughout active regions (Hu et al., 2015).

Hafnium oxide is a highly resistive material; its dielectric constant is several times higher than conventional silica gate dielectrics (Chen et al., 2010). The Pourbaix diagram shows the formation of a stable passive oxide on hafnium over the entire potential pH range (Pourbaix, 1974). Due to these features, thin layers of the oxide were applied for anticorrosion protection of stainless steel (304 SS) (Li et al., 2019) or magnesium alloys (Staišiūnas et al., 2020).

Hafnia films were obtained by various techniques such as RF and DC magnetron sputtering (Verelli and Tsoukalas, 2011; Dave et al., 2018), electrochemical anodizing (Fohlerova and Mozalev, 2019), electron beam evaporation (Ramzan et al., 2015), ultrasonic spray pyrolysis (Mendoza et al., 2010), UV-stimulated plasma anodizing (Kushitashvili et al., 2017), pulsed laser deposition (Plociennik et al., 2014), and electrochemical formation in organic electrolytes (Wang et al., 2017). Atomic layer deposition (ALD) is most widely used to form thin hafnia films and microstructures (Polydorou et al., 2018; Cui et al., 2017a; Singh et al., 2016; Oudot et al., 2018; Chen et al., 2010; Li et al., 2019; Zhang et al., 2017b; Ortiz-Dosal et al., 2017; Gaskins et al., 2017; Berdova and Liu, 2016). Thermal, mechanical, electrical, optical, and structural properties of ALD-formed HfO_2_ have recently been investigated and reviewed (Gaskins et al., 2017; Berdova and Liu, 2016; Jogiaas et al., 2015).

Little is known about photoelectrochemical (PEC) and protective properties of HfO_2_ films on Si in electrolytes. PEC studies are important for PV in many aspects. They provide information that correlates with that of the solid-state cells, so that there is no prior need to design a solar cell that characterizes Si surface photo-responsiveness (Cho et al., 2012; Ye et al., 2011). The PEC characterization can provide information about Si surface stability and its charging as well as the electron transfer from Si to electroactive species in the electrolyte—the process that is at the heart of PV devices for solar-driven hydrogen production. Here, we address the aforementioned questions performing the PEC measurements at open circuit and in wide range of cathodic polarizations in acid and alkaline mediums.

At the same time, the presence of electrolyte presupposes some complexity of the subject. In particular, the adsorption of hydrogen or hydroxide ions can influence the interfacial properties of the Si–oxide system. Distortion of oxide surfaces by penetration of electrolyte is a phenomenon of general nature (Perram et al., 1974). Water incorporation into the oxide layer could reduce its density, refraction index, or acoustic velocity (Davis and Tomozawa, 1995). Proton and water penetration into oxide is of importance to understand the instability of dielectrics during the operation of ion-sensitive field effect transistors (ISFETs). The presence of water or hydrogen-related species (–H, –OH) in ISFETs could enhance interface state generation and charge trapping (Topkar and Lal, 1995; Hazarika and Sharma, 2014). We studied here the electrolyte absorption by the hafnia film using mass change as a criterion. To that end, the quartz crystal nanobalance (QCN) was used, which provides information *in situ* and in real time with nanogram resolution.

## Experimental

P-type <100> silicon wafers from Si-Mat GmbH, Germany were used. Wafer diameter was 5 cm, the geometric area—22.8 cm^2^, the thickness – 275 ± 25 μm, and the resistivity was 10–20 Ω cm^−1^. The wafer surface was polished. The p-Si with thermal 10-nm SiO_2_ film was used as received from Si-Mat GmbH. XRD confirmed the amorphous structure of the silica layer.

HfO_2_ films were grown by ALD on the specimens 1 cm × 1 cm cut from the Si wafers. The ALD reactor Fiji F200 from Cambridge Nanotech has been used. The native oxide has been removed from the silicon surface using H_2_O_2_ + H_2_SO_4_ (1:1) and HF (1 HF: 40 H_2_O) solution. Tetrakis(dimethylamino)-Hf (TDMAH, [(CH_3_)_2_N]_4_Hf) from Strem Chemicals, Inc. and distilled water were used as hafnium and oxygen precursors. TDMAH and H_2_O were evaporated at 85 and 20°C, respectively. Argon was used as a carrier gas at a constant flow rate of 260 sccm. The HfO_2_ deposition temperature of 200°C and base pressure of ∼0.33 mbar were set in the deposition chamber. The deposition cycle consisted of a 0.06 s pulse of water, a 5 s purge, a 0.2 s pulse of TDMAH, and a 5 s purge. ALD is based on chemical self-limiting surface reactions, and the thickness of the coating is proportional to the number of cycles in the deposition sequence. Our estimations showed a linear dependence of the thickness vs. deposition cycles with a slope ∼0.13 nm/cycle within the 9–80 nm range. Basic principles of ALD were well reviewed (Miikkulainen et al., 2013; Schwartzberg and Olynick, 2015).

The thickness of the ALD hafnia layers was evaluated by the spectroscopic ellipsometry technique using a dual rotating compensator ellipsometer RC2 from J.A. Woollam, Co., Inc. The morphology and composition of the deposits were examined by SEM Helios NanoLab 650 from FEI equipped with an energy-dispersive X-ray spectrometer (EDX) from INCA Energy from Oxford Instruments.

Structural properties of the films were studied by the grazing incidence X-ray diffraction (GI-XRD) using a SmartLab diffractometer from Rigaku with an X-ray tube equipped with 9 kW Cu rotating anode. Grazing incidence geometry was used with the angle of the Cu Kα beam set to 0.5°, which enables investigation of thin films and reduces the influence of the substrate. A continuous scan mode was used in the 2Θ range from 15° ≤ 2θ ≤ 60° with a step of 0.02° and speed of 2.5°min^−1^.

The PEC measurements were carried out in an acid NaClO_4_ solution adjusted by hydrochloric acid to pH 3 and in NaOH diluted to pH 12. The measurements were conducted in a glass cell equipped with a quartz window using Luxeon 3W LED irradiation of *λ* = 505 nm wavelength and the energy density of 50 mW cm^−2^. Film transparency (*Tr*) has been evaluated using a reflectance calculator, which is based on the complex matrix form of the Fresnel equations (www.filmetrics.com/reflectance-calculator). Calculations at λ = 505 nm showed that HfO_2_ films of 10 nm in thickness are almost completely transparent (Tr = 0.972), while 60-nm films transmit about 67% of the light (Tr = 0.667).

**FIGURE 1:**
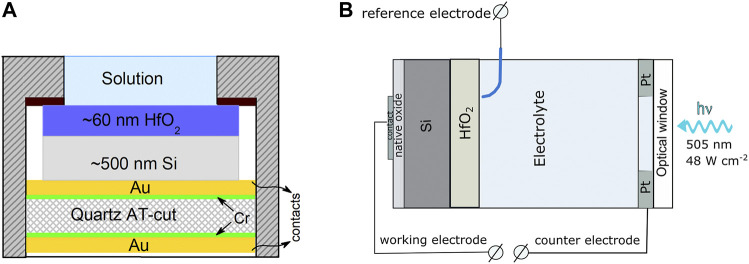
**(A)** Principal configuration of the QCN sensor, which is composed of Cr/Au/Si/HfO_2_ layers on quartz. Silicon layer was formed by RF magnetron sputtering, and HfO_2_ layer was formed by ALD; **(B)** experimental setup of the PEC measurements.

The measurements have been taken using PARSTAT 2273 equipment from Princeton Applied Research. The Hg/HgSO_4_ electrode in 0.5 M H_2_SO_4_ from International Chemistry Co., LTD was used as a reference electrode. The potential of this electrode was *E* ^0^ = 0.612 V vs. the normal hydrogen electrode (NHE) at 25°C, and the measured potentials all through the paper are given with respect to NHE. The counter electrode was a 4-cm^2^ Pt plate. The working electrode was mounted in an electrochemical glass cell so that the area of the electrode exposed to the electrolyte was 0.5 cm^2^ (Figure 1). The entire voltage applied to the cell comprised of several contributions: the drops in the electrolyte, within the hafnia film, the Si wafer, and the native silicon oxide in contact with the metallic conductor on the cell backside. The polarization curves were measured in a potentiodynamic mode at a potential scan rate of 0.5 mV s^−1^. Mott–Schottky plots were obtained at a frequency of 1 kHz using the PARSTAT 2273 equipment.

The QCN measurements were performed at ambient temperature (20 ± 2°C) using the P/G/FRA system Autolab 302 from Metrohm AG, equipped with the Quartz Crystal Microbalance QCM 922 unit from Princeton Applied Research. AT-cut quartz resonators with Au electrodes produced by Intellemetrics Global Ltd were used to prepare the quartz crystal nanogravimetry sensors (Figure 1A). Si layer has been sputtered on the Au sublayer using a Univex 350 from Leybold Vacuum GmbH. As a sputtering target, the p-Si wafer was used. Before deposition, the sputtering chamber was evacuated to ∼2 × 10^–4^ Pa pressure and filled with Ar (99.999%). The working pressure of the chamber was maintained at 0.19 Pa, and the substrate temperature was set at 50°C. The sputtering time was 15 min, and the sputtering power was 25 W, which yielded a coating of ∼0.2 μm thickness. The HfO_2_ layer on the Si surface was deposited by ALD, as described before. The fundamental frequency of the sensor was *f*_0_ = 6 MHz, and the sensitivity factor was *K* = 12.4 ng Hz^−1^ cm^−−2^. The principal configuration of the mass sensor and the electrochemical cell is shown in Figure 1.

The morphology and composition of the deposits were examined by a SEM Helios NanoLab 650 from FEI equipped with an energy-dispersive X-ray spectrometer (EDX) from INCAEnergy from Oxford Instruments.

Raman spectra were recorded using a confocal spectrometer in *Via* (Renishaw, United Kingdom) equipped with a thermoelectrically cooled CCD detector (−70°C) and a microscope. Raman spectra were excited with 532-nm radiation from the CW diode-pumped solid-state (DPSS) laser (Renishaw, United Kingdom). The laser power at the sample was restricted to 0.6 mW. The 50x/0.75 NA objective lens and 1800 lines/mm grating were used to record the Raman spectra. The overall integration time was 800 s.

## Results and Discussion

### Structural Study

**FIGURE 2:**
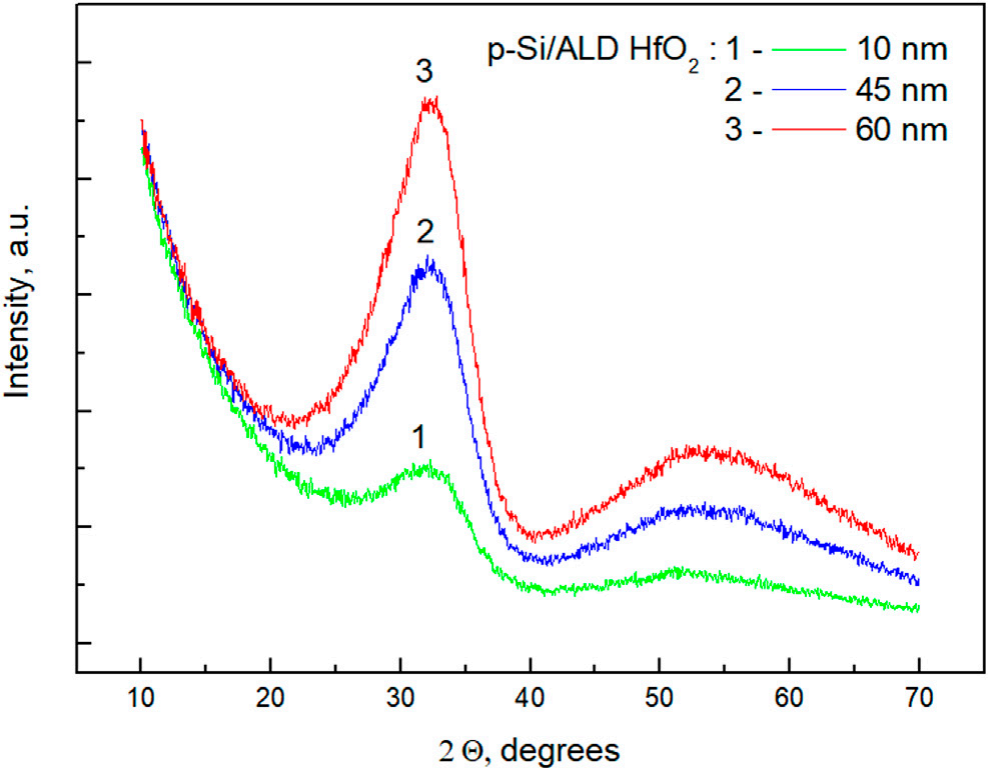
GI-XRD patterns obtained for p-Si with ALD deposited HfO_2_ layers of different thickness: 10, 45, and 60 nm.

The structural properties of the ALD HfO_2_ films were studied by GI-XRD (Figure 2). The grazing incidence geometry of this method enables investigation of the crystallographic structure of thin films and reduces the influence of the silicon substrate. The GI-XRD provided reliable structural data of HfO_2_ and ZrO_2_ thin films from ∼9 to ∼30 nm in thickness (Park et al., 2017; Sharma et al., 2017).

The XRD data do not indicate crystallinity features of the films—a broad peak at 2Θ ∼ 33° indicates an amorphous structure. A similar peak was observed for amorphous HfO_2_ nanoparticles with diameter ∼4 nm prepared by the ammonia catalyzed hydrolysis and condensation of hafnium (IV) tert-butoxide (Chaubey et al., 2012). The authors showed that the amorphous structure did not change during annealing of the samples up to 400°C; indications of monoclinic HfO_2_ phase appeared at 500 °C or higher. Our study showed appearance of HfO_2_ crystallinity with a monoclinic structure when coating thickness has been increased to 80 nm (Staišiūnas et al., 2020).

Crystallization phenomena of HfO_2_ nanofilms have attracted considerable attention (Cui et al., 2017b; Singh et al., 2016; Kim at al., 2003; Fan et al., 2012; Zhang et al., 2019). The crystallinity of the films is important in terms of Si surface passivation. The films on silicon up to 35 nm in thickness were found predominantly amorphous with minor localized nucleation of crystals (Cui et al., 2017b; Singh et al., 2016). The amorphous structure yielded highly effective electronic passivation, whereas the film crystallization achieved by annealing at higher temperatures led to degradation of the effective surface recombination velocity. Thickness-dependent crystallization was also studied for the films obtained by ion-assisted RF deposition (Zhang et al., 2017)*.* The authors observed the formation of grains when the coating thickness was greater than 12 nm. The grain size and density increased with the coating thickness. This phenomenon was explained in terms of the Gibbs energy change of the atom clusters. The crystallization depends on the ratio between the cluster surface area and its volume. Thinner coatings have a smaller cluster size and a higher surface-to-volume ratio; this results in a positive change in Gibbs energy and suppression of the amorphous-to-crystalline transformation. In the present study, such suppression is likely due to the deposition of extremely thin single layers using a layer-by-layer ALD approach (∼0.13 nm/cycle). The produced films, therefore, did not show appreciable crystallinity in the studied thickness range.

### Nanogravimetry and Raman Spectroscopy

**FIGURE 3:**
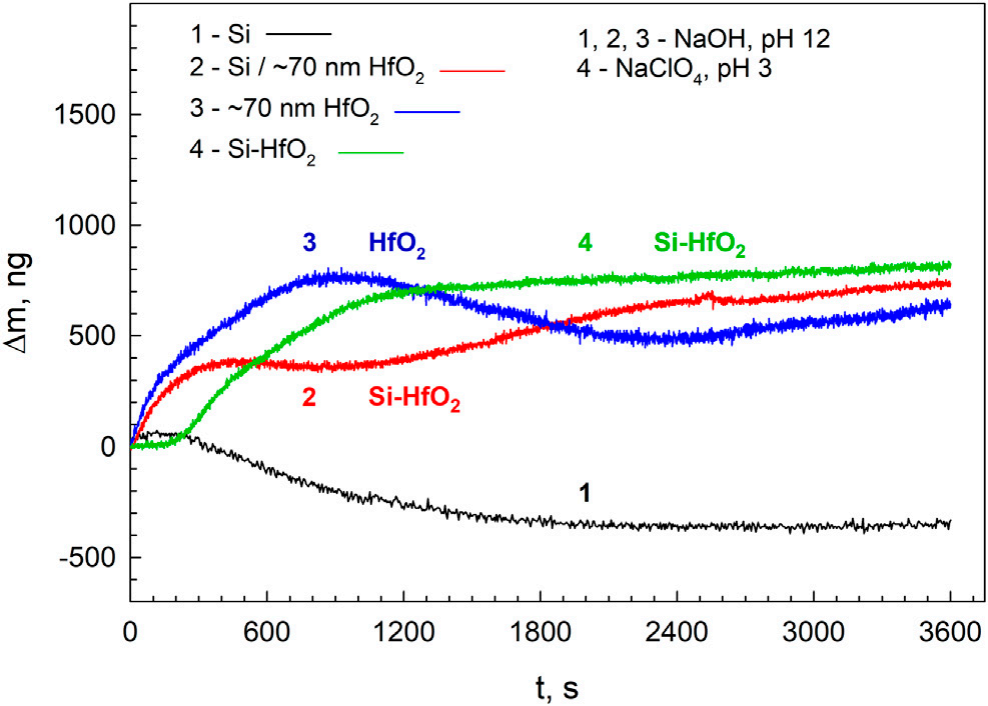
Mass change determined by the QCN of the samples with 70-nm HfO_2_ layer on Si (2, 4) and Au (3) substrates when exposed in NaOH (pH 12) (1–3) and 1 M NaClO_4_ adjusted to pH 3 (4). Curve 1 shows the mass change of hafnia-free silicon pretreated in an HF solution.

Nanogravimetry provides sensitively the data on the instability of Si–HfO_2_ electrode, which results in a change in electrode mass (Figure 3). The measurement shows *in situ* and real-time mass change with nanogram resolution immediately after the sample comes into contact with the electrolyte.

The nanogravimetric data obtained for the Au–HfO_2_ electrode indicate an increase in electrode mass during the exposure. With time, the mass becomes more or less constant for all electrodes. Note that the mass change of the Au–HfO_2_ electrode does not differ greatly from that of the Si–HfO_2_ electrode. Due to the chemical inertness of the Au substrate, the mass gain is attributable to the events within the oxide film, most likely, the electrolyte absorption by the film. The mass of the film increased on average by up to Δm ∼ 700 ng cm^−2^ during 1 hour of exposure. An increase in the mass of the film on the Si substrate was of similar value. There were no indications observed of oxide dissolution, which would be detected as a decrease in electrode mass. This implies effective electrode protection by the hafnia film both in acidic and alkaline media. As commonly known, silicon is stable in water and acidic solutions due to the formation of a stable protective oxide. This oxide, however, is unstable in alkaline solutions. Chemical and electrochemical corrosion could contribute to the mass effect in alkaline solutions (Hu et al., 2015). The mass decrease in the electrode unprotected with a hafnia coating indicates the corrosion process. With time, the dissolution ceases due to surface passivation by corrosion products.

It is also important that the mass curve for the Si–HfO_2_ electrode in acid solution does not differ greatly from that in alkaline solution (Figure 3). Thus, the amount of the absorbed electrolyte does not depend on solution acidity, and the mass effect is similar as the densities of both electrolytes do not differ considerably. The mass of the HfO_2_ layer 70 nm in thickness is calculated to be *m* = 63 μg cm^−2^ assuming its density, *d*_HfO2_ = 9.68 g cm^−3^. The electrolyte intake is Δ*m* ∼ 0.7 μg cm^−2^; this corresponds to ∼4.4% of the initial oxide mass. The volume of accommodated solution will make up approximately ∼50% of the initial oxide volume.

The QCN data show that the electrolyte penetrates the thin oxide film and transforms it to a hybrid electrode Si–HfO_2_–electrolyte. The electrolyte plays an essential role in interfacial charging.

The question also arises about perchlorate penetration into the oxide layer, first of all, due to the presence of non-uniformities and defect sites in the surface layer. Raman spectroscopy implied that perchlorate ions do not enter appreciably the oxide layer during illumination; they are retained at the oxide/electrolyte boundary. This method is applied for effective detection and discrimination of oxyanions. Perchlorate possesses a relatively high Raman cross section (12.7 × 10^–30^ cm^2^ sr^−1^ molecule^−1^) (McCreery, 2002) and can be determined by normal Raman as well as surface-enhanced Raman spectroscopy (SERS) approaches (Hao and Meng, 2017). The detection limits range from micromolar to nanomolar levels (10^–6^–10^–9^ M; 0.1 mg L^−1^–0.1 μg L^−1^). To that end, various SERS substrates mainly based on gold and silver are used: functionalized gold–silica (Au–SiO_2_) (Wang et al., 2006), 2-dimethylaminoethanethiol (DMAE)-modified gold nanoparticles (Gu et al., 2009), silver, gold, silver–gold, and selective sorbents (Wang and Gu, 2005; Nuntawong et al., 2013; Gu et al., 2004; Byram et al., 2018; Jubb et al., 2017). Also, the discrimination of perchlorate from salt mixtures has been demonstrated by Raman spectroscopy (Zapata and Garcia-Ruiz, 2018).

**FIGURE 4:**
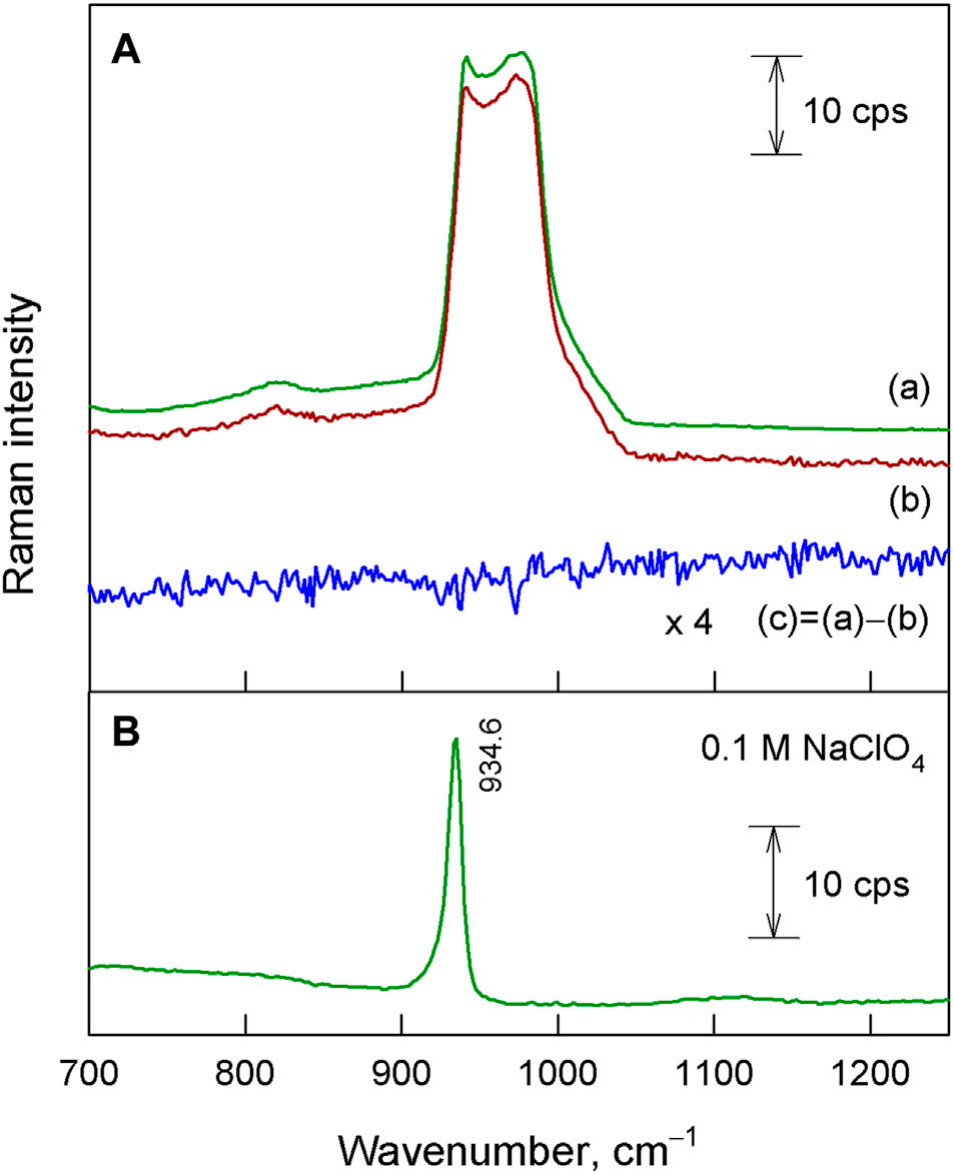
**(A)** Raman spectra of Si–HfO_2_ treated with radiation in NaClO_4_ solution (a) and Si reference sample (b). Difference spectrum (c) with a magnified intensity (×4) is also shown. Spectra are shifted vertically for clarity. **(B)** Raman spectrum of 0.1 M NaClO_4_ aqueous solution. Excitation wavelength is 532 nm.

Figure 4 compares the Raman spectrum of a radiation-treated Si–HfO_2_ sample with the spectrum of a Si reference. P-type Si with a 60-nm HfO_2_ layer has been pre-exposed in 1 M perchlorate electrolyte for 5 min, illuminated for 5 min by λ = 505 nm light, and withdrawn from the electrolyte. The broad and intense band between 920 and 1,050 cm^−1^ belongs to the second-order Raman spectrum of the silicon substrate ( Gillet et al., 2017 ). Observed spectral features are very similar with silicon substrate Raman spectrum. The Raman spectrum of 0.1 M NaClO_4_ aqueous solution (Figure 4B) exhibits a well-defined band at 934.6 cm^−1^ associated with the symmetric stretching vibration of the ClO_4_^−^ ion (Zdaniauskienė et al., 2018; Niaura and Malinauskas, 1998). To enhance small spectral changes, we have constructed a difference spectrum. However, this spectrum does not indicate the presence of any additional bands compared with the silicon substrate. Thus, the Raman spectroscopy study does not provide any evidence confirming the presence of ClO^4−^ ions in the studied Si–HfO_2_ structure. Thus, one could conclude that the mass gain observed by the QCN (Figure 3) was due to the incorporation of water along with protons and hydroxyl. Protons could increase the state density at the Si/oxide interface, and such modification could form some areas of an interconnected network of defective regions (Topkar and Lal, 1995). Penetration of water could create increased mobility paths for the protons (Topkar and Lal, 1995).

### Photoeffects

The PEC measurements have been performed using *λ* = 505 nm illumination, which is around the middle of the visible spectrum (∼380 to ∼750 nm). The applied photon energy (2.45 eV) is greater than the bandgap energy of Si (*E*_g,Si_ = 1.1 eV); it was sufficient to excite the Si photoelectrons to the conduction band. Note that the bandgap of ALD HfO_2_ films under dry conditions is much higher (*E*_g,HfO2_ = 5.9 ± 0.5 eV) (Cheynet et al., 2007). The oxide can retain its insulating properties in electrolyte as well, particularly, at early stages of exposure.

**FIGURE 5:**
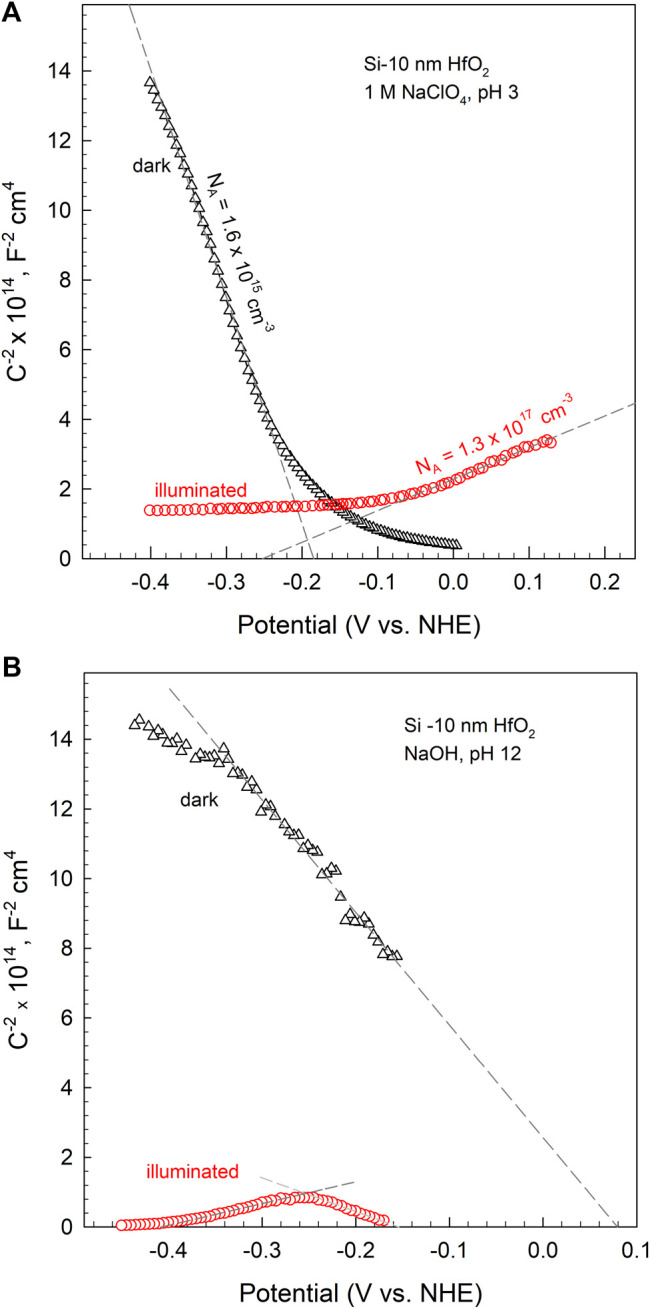
Mott–Schottky plots for p-Si with 10-nm HfO_2_ layer in the dark and under illumination (*λ* = 505 nm, *N* = 50 mW cm^−2^) in **(A)** 1M NaClO_4_ at pH 3 and **(B)** NaOH at pH 12. The electrode potential has been stepped from the open circuit in negative direction.

Figure 5 shows a measurement of the electrode capacitance (*C*) depending on the electrode negative polarization (-Δ*E*). The data are displayed as *C* ^−2^ vs. *E* plots that are in accordance with the Mott–Schottky relation:

$$C_{sc}^{-2}=\frac{2}{\varepsilon_{0}\varepsilon N_{D/A}e}\left(E-E_{fb}-\frac{kT}{e}\right)$$ (1)

where *C*_SC_ stands for space charge capacitance, *N*_D/A_ means the number of donors or acceptors in the space charge layer, *k* is the Bolzmann’s constant, *ε* is the dielectric constant of the semiconductor, *ε*_0_ is the permittivity of free space, *T* is the absolute temperature, *e* is the charge of a hole or electron, and *E*_fb_ is the flatband potential. *E*_fb_ means the potential, at which the energy band bends to zero. This parameter can be determined when extrapolating the *E*–*C* ^−2^ curve to *C* ^*−2*^ = 0. The curve slope reflects the doping level of the dark electrode or the density of the photogenerated charges in the space charge region.

**FIGURE 6:**
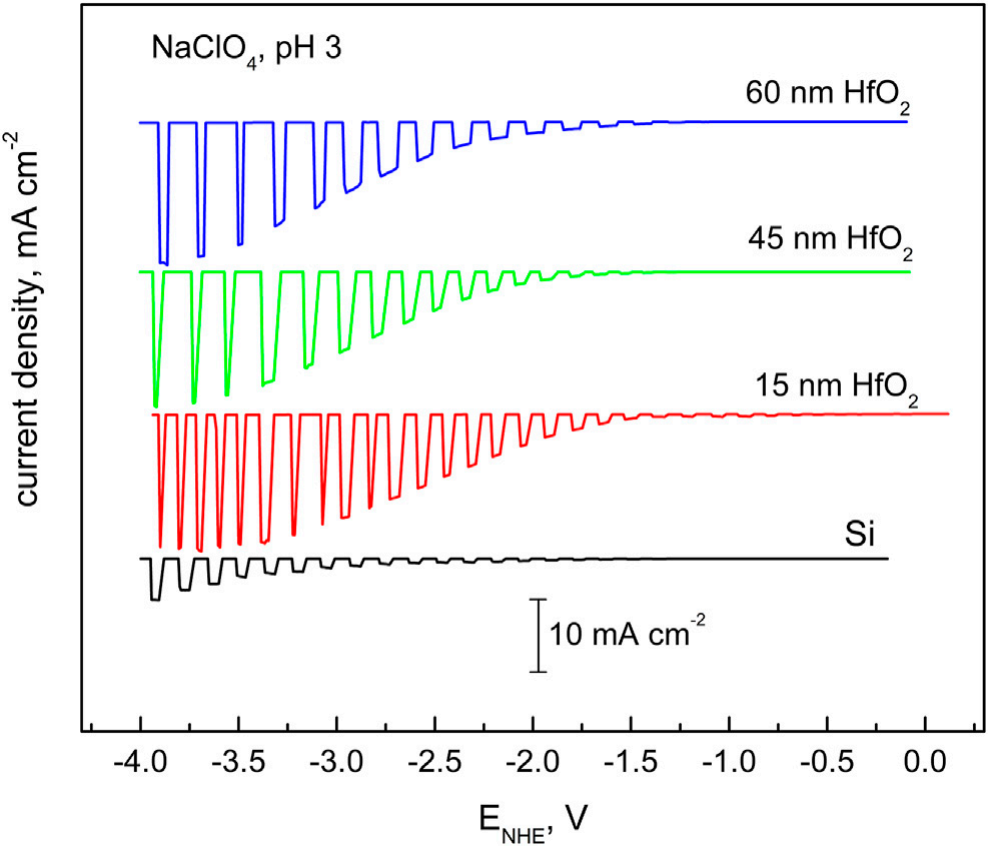
Cathodic photocurrents of hydrogen reduction over wide region of cathodic voltages obtained for *p*-Si–H electrode prepared by treatment in HF solution (marked as Si) and that coated with HfO_2_ layer with different thickness. Electrolyte: 1 M NaClO_4_ (pH 3). The illumination of 505-nm wavelengths and intensity of 50 mW cm^−2^ have been chopped at certain intervals. The potential sweep rate was 5 mV s^−1^.

**FIGURE 7:**
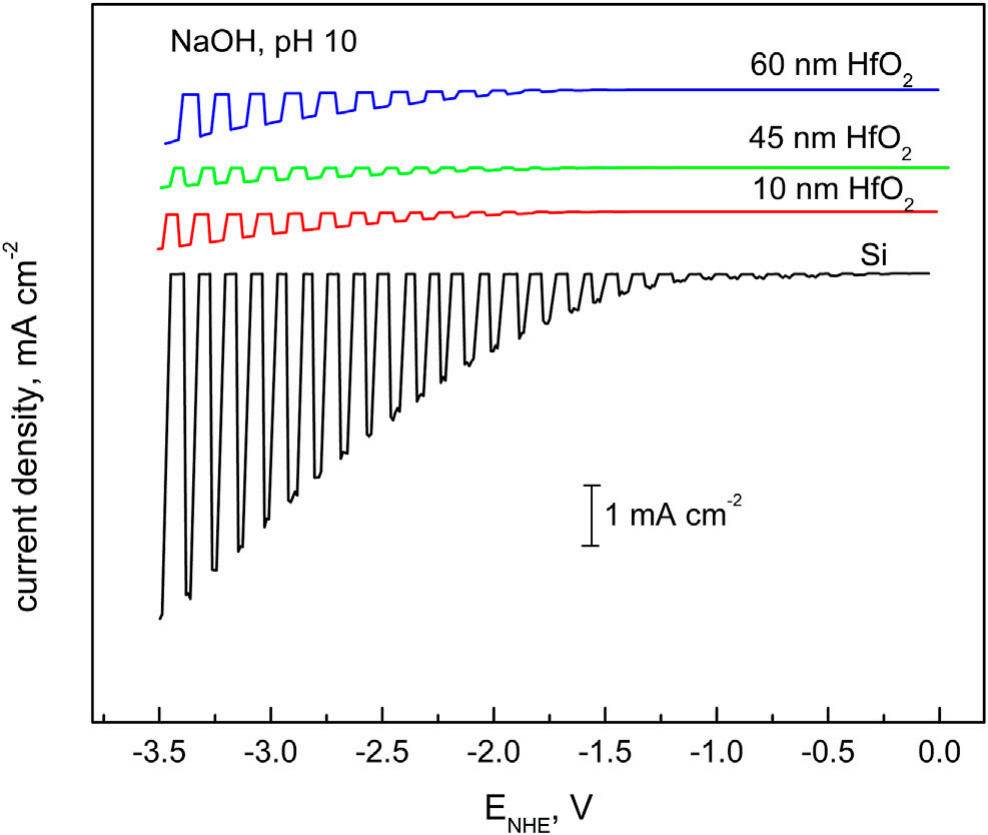
Cathodic photocurrents of hydrogen reduction over wide region of cathodic voltages obtained for *p*-Si–H electrode prepared by treatment in HF solution (marked as Si) and that coated with the HfO_2_ layer with different thicknesses. Electrolyte: NaOH solution at pH 12. The illumination of 505-nm wavelengths and intensity of 50 mW cm^−2^ have been chopped at certain intervals. The potential sweep rate was 5 mV s^−1^.

The electrodes in the studied potential range (Figure 5) were nearly ideally polarizable; no appreciable Faradaic process was observed under dark and illuminated conditions in this region, as Figures 6, 7 demonstrate. The capacitance values of the dark electrode were on the order of nanofarads (nF cm^−2^). Such low values indicate that the capacitances are attributable to the solid phase (e.g., space charge region), rather than the solid–solution interface. A double layer, which is formed by water and electrolyte ions (Helmholtz layer), typically has a capacitance on the order of microfarads (μF cm^−2^) (Bard and Faulkner, 2001; Zhang, 2004). As commonly known, *C* is in inverse proportion to the distance between positively and negatively charged zones. The space charge region can extend up to 2000 Å, while the Helmholtz layer is only several angstroms wide (Bard and Faulkner, 2001; Zhang, 2004). The lowest *C* value will predict the total capacitance (in analogy with the equivalent capacitance (*C*_eg_) with actual capacitances (*C*_1_, *C*_2_) connected in series: 1/C_eq_ = 1/C_1_ + 1/C_2_).

In an acid solution, a negative potential shift needs to be exerted for the dark electrode to achieve *E*_fb_, that is, to make the energy distribution flat (Figure 5). This means that the dark silicon surface is positively charged. In an alkaline solution, a positive potential is necessary to apply to obtain the energy flatness, and this means the silicon surface is negatively charged. These data imply that the Si–HfO_2_ electrode interfacial charge depends on solution pH. The Si surface charge is determined by the specific adsorption of hydrogen or hydroxide ions. Adsorption of these ions determines the potential of the Helmholtz layer, which obeys the relation Δ*E = B* – 0.059 pH, that is, potential varies by –59 mV per pH unit according to thermodynamic assumptions [(Zhang, 2004), P. 13]. When the oxide film is present, the ions can penetrate the HfO_2_ film and charge the interface positively (pH 3) or negatively (pH 12).

The capacitance of the dark electrodes (*C*_D_) decreases with the negative potential shift (-Δ*E*). Such feature is characteristic of a p-type semiconductor where holes (*h* ^+^) are major carriers. The number of positive charges decreases with -Δ*E* and, consequently, *C*_D_ also decreases.

The curve slope in Figure 5 gives the carrier number *n* = 1.4 × 10^15^ cm^−3^, which does not differ greatly from the value calculated for the sample bulk using the wafer conductivity as a criterion (Klaassen, 1992) (*R* = 10 Ω cm^−1^ and *n* = 1.36 × 10^15^ cm^−3^).

Under illumination, the capacitances (C*) increase to the level of several hundreds of nanofarads (e.g., 170–220 nF cm^−2^ in Figure 5). A negative bias (*E* ∼ −0.25 V) needs to be applied in acid solution to attain the energy flatness; this indicates the illuminated surface is charged negatively by photoelectrons. The C* value increases with −Δ*E*. The light generates minority carries—electrons, the number of these increases with −Δ*E* and, consequently, C* also increases. The curve slope in Figure 5 indicates the number of charge carriers in the space charge region of the illuminated electrode to be *n* ∼ 1.16 × 10^17^ cm^−3^. This number is significantly higher than that determined in darkness. Note also that crystalline Si contains ∼5 × 10^22^ atoms per cm^3^ (Bard and Faulkner, 2001; Zhang, 2004).

The shape of the *E*–C* curve in alkaline solution is more complicated: it shows an inversion with characteristic ascending and descending slopes (Figure 5B). The ascending part indicates that Si surface is charged negatively, whereas the descending part implies a positive charge, as has been observed in the acid solution. Different capacitive behavior in acid and alkaline solutions implies different structural properties of the interface on the solution side. As discussed before, in an alkaline solution, the illuminated Si surface will be charged negatively by adsorbed OH^−^ ions. Negative polarization will diminish the capacitance because the supplied electrons will reduce the number of positive charges. However, at the same time, electrolyte Na^+^ ions will be attracted to the interface due to Coulomb interaction. The C* will start to increase when Na^+^ becomes dominant at the surface, as the curve in Figure 5B shows. It is worth mentioning that an interface layer model composed of the surface complexes ≡SiOH, ≡SiO-Na^+^, and ≡SiO- has been proposed (Zhang, 2004; Dove and Elston, 1992).

Figures 6, 7 characterize hydrogen reduction, that is, electron transfer through the hafnia film in acid (Figure 6) and alkaline (Figure 7) solutions. The effect of hafnia film in both solutions is different. In acid solutions, the film enhances the reduction process when compared to that of the coating-free sample. In contrast, in alkaline solutions, an inhibition effect is observed. The acceleration effect implies protection from corrosion and prevention of the oxide layer formation, which has a higher passivating capacity than that of HfO_2_. Si passivation may occur under acidic or neutral conditions. It is also important that corrosion (oxide formation) could occur even at cathodic polarizations due to the chemical (not electrochemical) nature of the process (Hu et al., 2015). Silica is not stable in an alkaline environment; if formed, it dissolves, exposing the electrode surface to corrosion. Hafnia film in such a case plays a protecting role, at the expense, however, of electrode activity.

The discussed situation is rather typical: while mitigating corrosion, the oxide films reduce surface activity, and the electrode becomes electrochemically less active in certain potential regions. The dilemma is typically solved in favor of passivity because dissolution leads to continual destruction of the semiconductor and failure propagation throughout active regions (Hu et al., 2015). One can conclude that hafnia films on silicon showed stability and effective protective properties both in alkaline and acid environments.

## Conclusion

Ultrathin films of hafnium oxide in the range of 15–70 nm were deposited by the ALD on p-Si (100). XRD identified the amorphous structure of the films.

The QCM measurements with HfO_2_ films on Si and Au substrates indicated the dynamics of the electrolyte intake into the oxide film. No indications of oxide dissolution have been observed in acidic and alkaline electrolytes. The mass gain effect did not depend on electrolyte acidity; similar mass gain was observed at pH 3 and 12. Raman spectroscopy indicated that perchlorate does not enter the hafnia film appreciably.

Mott–Schottky plots showed that the dark Si surface adjacent to the Si–HfO_2_ interface is positively charged in acid solution and negatively charged in alkaline solution. The number of photoelectrons was determined to be much greater than the doping level of the p-Si.

The cathodic photoactivity of the p-Si electrode protected by HfO_2_ film was studied with respect to the reaction of hydrogen reduction in acid and alkaline solutions. In acid solution, the film enhanced the reduction process when compared to that on the coating-free electrode. The acceleration effect was explained in terms of the prevention of silicon oxide formation. In an alkaline electrolyte, an inhibition effect of the film was determined. Hafnia films protected Si from corrosion in this medium; however, at the same time, the film reduced electrode activity.

## Data Availability

The original contributions presented in the study are included in the article, further inquiries can be directed to the corresponding author.
